# Dual Behavioral–Physiological Buffering of Mothers' Milk Facilitates Drought Adaptability of Pastoralists and Agropastoralists in Northern Kenya

**DOI:** 10.1002/ajhb.70057

**Published:** 2025-05-19

**Authors:** M. Fujita, K. Wander, B. Straight, G. Wamwere‐Njoroge

**Affiliations:** ^1^ Department of Anthropology Michigan State University East Lansing Michigan USA; ^2^ Biomarker Laboratory for Anthropological Research Michigan State University East Lansing Michigan USA; ^3^ Department of Anthropology Binghamton University (SUNY) Binghamton New York USA; ^4^ Laboratory for Anthropometry and Biomarkers Binghamton University Binghamton New York USA; ^5^ Department of Gender and Women's Studies Western Michigan University Kalamazoo Michigan USA; ^6^ International Livestock Research Institute Marsabit Kenya

**Keywords:** drought adaptability, folate deficiency, mothers' milk, protein‐energy malnutrition, vitamin A deficiency

## Abstract

**Background:**

Mothers physiologically buffer key milk nutrient content against nutritional stress. How this is nested in upstream behavioral buffering is not well understood.

**Objectives:**

The study explored whether pastoralists and agropastoralists' economic or other behavioral coping strategies against droughts, such as livestock sales and child fosterage, influence maternal risk for malnutrition or milk nutrient content.

**Methods:**

Using data from 221 breastfeeding mothers in drought‐stricken northern Kenya, we estimated generalized structural equation models to evaluate pathways linking behavioral coping variables to maternal malnutrition—underweight, vitamin A deficiency (VAD), and folate deficiency (hyperhomocysteinemia)—and to milk energy, retinol, and folate content directly or mediated by maternal malnutrition. Predictors of interest included land size, proportion of cattle/goat herds sold, children fostered/adopted out, and children living at home. Akaike Information Criterion guided model fit assessment.

**Results:**

Land size was positively associated with maternal underweight and VAD. Child fosterage and cattle sold were inversely associated with underweight, while child fosterage and goats/sheep sold were positively associated with hyperhomocysteinemia. Children living at home were inversely associated with VAD, particularly with larger land size, and positively associated with milk retinol. Milk folate was positively associated with hyperhomocysteinemia.

**Conclusions:**

Behavioral buffering strategies, such as fostering out children, offer incomplete protection against maternal malnutrition. The lack of effects of investigated behavioral buffering strategies on milk variables suggests physiological buffering closes the gap left by incomplete behavioral buffering. Dual behavioral‐physiological buffering facilitates the drought adaptability of agropastoralists, yet heavy reliance on physiological buffering for micronutrients suggests high maternal cost.

## 1 Introduction

East African pastoralists and agropastoralists, particularly those in the Horn of Africa, the arid peninsular region at the easternmost end of the continent, are affected by increasingly severe droughts. Unfortunately, these droughts are projected to become more frequent and extreme in the next decades (Haile et al. 2020). In Kenya alone, an estimated 9 million people depend on pastoralism today, and in northern Kenya, pastoralists face drought‐related livelihood risks, compounded by increased encroachment of external interests such as wildlife conservancies and large‐scale wind and oil projects (Schilling and Werland 2023).

Prolonged droughts can lead to livestock losses as animals succumb to starvation and diseases, and ultimately, to food shortages for households. Pastoralists cope with droughts in many ways. Maintaining animals' health is difficult without reliable water and vegetation, so selling animals is a viable strategy to minimize economic loss. Another strategy is to keep animals, risking that animals may be lost or may become too thin to sell. Taking animals to places where microclimates foster more vegetation feeding opportunities for animals, provided that families have adequate labor and expertise, can decrease the risk associated with keeping animals. In dry weather conditions, animal milk production reduces to such low levels that pastoralists and agropastoralists have to increasingly consume cereals and pulses acquired by proceeds from selling goats, sheep, and cattle (Fratkin et al. 1999).

In addition, pastoralists employ other strategies such as diversifying economic activities, including fetching and selling firewood and adopting plant cultivation where it is an option (Mburu 2017), resorting to negative coping mechanisms like charcoal burning (Fratkin 2013; Little et al. 2001), and adjusting household composition and work allocation (Fratkin et al. 1999; McCabe 1985; McCabe et al. 1988; Roth 1990, 1991). Delegating livelihood tasks to children can enable parents to search for food. Conversely, sending children to live with relatives, including those who need labor for livestock herding, can not only help the relatives manage their livestock wealth (Mburu 2017) but also decrease the household size and therefore food needs in the face of food shortages. Having fewer household members, particularly children, whom adults typically spare from reduction of meals as much as possible, can bring a relief to the household's food constraints. Fostering children out may benefit children in terms of their nutrition, health, or educational opportunities (Archambault and de Laat 2010; Schrijner and Smits 2018; Shell‐Duncan 1994) although the historic and social contexts of the communities and/or household characteristics may diminish or nullify such benefits (Hampshire et al. 2015; Lawson et al. 2017).

In northern Kenya, there have been efforts to encourage pastoralists to settle and engage in crop farming, especially after droughts. Families who settle in this way tend to want to continue to keep livestock, both for social capital and economic insurance, given the risk of relying on plant cultivation in this drought‐prone area, although the risk can be partially managed by technological solutions such as drought‐tolerant cultivars. Households in settled communities and those who continue nomadic lifeways maintain ties through marriages and age‐set rites, and often exchange grain, livestock, and labor; therefore, settled and nomadic pastoralists are integrated in ethnic identity and economic system (Little 1983; Spencer 1998).

Despite this mixed strategy, those dependent on crops are most vulnerable to drought (Marsabit County Government 2018). From a human adaptability perspective (Leonard 2018; Moran 2022), settled agropastoralists' continued investment in livestock herds constitutes a behavioral buffer against adverse impacts of drought‐related ecological stress on their economic health. Herds, through geographic mobility and dispersal, can better tolerate droughts than agricultural crops. Animals can be sold more easily than land plots to dampen economic loss. Investment in herds and flexibly managing livestock size, composition, and geographic mobility of livestock and children in cooperation with multiple families are sound coping strategies in this setting.

Severe droughts would test the limits of behavioral buffers, and it might be expected that vulnerable segments of the population, such as lactating mothers and children, experience substantial nutritional stress at these times.

Lactating mothers' physiology generally buffers their milk nutrient content to protect infants. Previous research has described some of this maternal buffering in the form of null associations between maternal condition (such as iron‐deficiency anemia, other anemia, and inflammation) and human milk nutrient content (protein, lactose, or fat), or even elevation of milk folate content among folate deficient mothers in agropastoral communities of northern Kenya experiencing a severe drought (Fujita et al. 2019a, 2019b, 2022). However, the understanding of maternal physiological buffering of human milk has so far been largely detached from the investigation of upstream economic and other coping behaviors, such as livestock herd management and the movement of children between households (Fratkin and Roth 1990; Roth 1990; Talle 2004).

**FIGURE 1:**
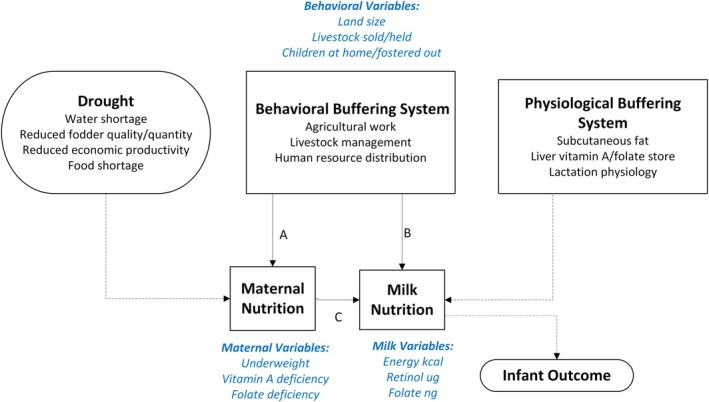
Conceptual framework for the dual buffering system. The dual buffering system consisting of economic/behavioral buffering and physiological buffering concurrently and interactively dampening adverse impacts of drought‐related ecological stress. The present study modeled pathways denoted as A, B, and C, applying generalized structural equation modeling. Dashed arrows indicate inferred pathways. List of variables utilized in the present study are shown in blue.

Here, we use a quantitative approach to assess economic coping behaviors as the first layer of buffer for maternal condition against the harsh stress of drought, and maternal physiological buffering of milk as the second layer using the conceptual framework shown in Figure 1. The purpose of this study was to explore whether and how economic behavioral strategies such as land holding, livestock sales, and child fosterage influence maternal malnutrition (path A) and milk nutrient content (path B). Evaluating these pairs of associations—first between economic/behavioral buffers and maternal nutrition and then between economic/behavioral buffers and maternal milk content—allows us to assess concordance (i.e., is an economic buffering variable associated with both maternal nutrition and milk content?) to infer the relative extents of behavioral buffering and maternal physiological buffering. If we observe similar associations between economic/behavioral buffers and maternal nutrition and between economic/behavioral buffers and milk content, and a pathway between maternal nutrition and milk content, that points to the importance of behavioral buffering. If, on the other hand, we observe that these economic/behavioral buffers have divergent associations with maternal nutrition and milk content, with little evidence of a pathway through maternal nutrition to milk content (path C), that points to an important role for physiological buffering.

## 2 Methods

### 2.1 Data Source and Context

The study team conducted a secondary analysis of retrospective interview data (available at https://zenodo.org/records/13285825) on household behavioral variables and biomarkers of maternal and human milk nutrition from breastfeeding mothers in Ariaal agropastoral communities of northern Kenya, surveyed during a prolonged drought in August 2006. Key variables of interest utilized in the present analysis are listed in Figure 1. The survey (Fujita 2008) was approved by the institutional review boards of the University of Washington and Kenya Medical Research Institute. No further approvals were necessary for the present study of de‐identified data.

The study area, spanning from the mid‐slope of Mount Marsabit (1700 m) to lowland arid plains, has been classified as one of the most arid regions in Kenya with erratic and unreliable rainfalls (800–1000 mm in the highlands and 200–250 mm in lowlands) and scarce access to water and pasture (Hazard and Adongo 2015; Marsabit County Government 2018). The survey focused on two highland communities (agropastoralists) in Karare and Kituruni, and one lowland desert community (pastoralists) in Korr. There were no households with piped water or public electricity.

The historical, sociocultural, and ecological contexts of settled Ariaal and the closely related Rendille and other pastoralists have been described in depth elsewhere (Fratkin 2004, 2019; Fratkin and McCabe 1999; Fratkin and Roth 1990; Fratkin and Smith 1995, 2005; Roth and Fratkin 2005). Briefly, during the severe droughts of the 1970s, many herders in northern Kenya lost livestock to disease and became destitute. Around Marsabit Mountain, some of these families took up agriculture for subsistence and cash cropping as part of economic development projects. After settlement, they began to invest in livestock whenever possible to rebuild small herds. Others settled in and around drought‐relief distribution stations to receive food aid and/or to seek other economic opportunities of sedentary communities (Fratkin 2004; Fratkin and Smith 1995; Roth 1999). The mother‐infant/child nutritional and health characteristics of these communities have also been described elsewhere (Brunson et al. 2009; Fratkin et al. 1999, 2004; Fujita, Lo, et al. 2012; Fujita et al. 2004, 2024, 2012a, 2012b, 2005; Fratkin 2004; Fujita and Wander 2025; Miller 2011; Nathan et al. 1996; Paredes Ruvalcaba et al. 2020; Vankayalapati et al. 2024).

In northern Kenya, nearly all mothers breastfeed, and formula use is extremely rare. The Demographic Health Survey (Kenya National Bureau of Statistics 2015) reports breastfeeding rates of 98.8%, complemented by animal milk in varying rates depending on postpartum time (e.g., < 4% for neonates and 10%–30% for infants of 1–5 months; Eastern Province). The introduction of solid foods begins around 6 months, and the weaning transition continues until the child is 2 years or older, with the median duration of any breastfeeding 24.5 months and the median birth interval 34.9 months (Kenya National Bureau of Statistics 2015). Simultaneous breastfeeding of multiple children is likely, but the rates are unknown.

At the time of the survey, after multiple years of extreme aridity (seasonal rainfalls partially or fully failed in 2004 and 2005), northern Kenyan pastoralist and agropastoralist households faced dramatic nutritional stress, and the fodder and livestock conditions were also nearly the worst of the decade leading into the 2006 drought (Marsabit District 2006; USAID 2018; World Health Organization 2006). The adversity was compounded by high commercial food prices, inter‐tribal livestock raids, and slow mitigation by government and aid agencies (Marsabit District 2006; USAID 2018; World Health Organization 2006).

### 2.2 Variables

Survey interviews included household economic variables such as land size, livestock number, and species held at the time of the interview, and whether they sold any livestock in the preceding year. Also, mothers were asked to provide their reproductive history, including all children born (alive or not) and whether living children reside with the mother or elsewhere at the time of the survey. Maternal and human milk nutritional variables were available from our previous research (Fujita 2008; Fujita et al. 2017; Fujita et al. 2019a, 2019b; Fujita et al. 2022).

The study used household land size (in acres) to characterize participating households' investment in agriculture and tropical livestock unit (TLU) to characterize their livestock holdings. TLU is a composite measure to describe the size of mixed‐species livestock. TLU was calculated as: TLU = 1.0 camel + 0.8 cattle + 0.10 small goats and sheep (FAO 1967; Roth 1990).

For livestock sales, categorical variables, cattle sold and goats/sheep sold were derived, each having four categories: (1) had no animals to sell (i.e., no option to sell), (2) > 50% sold, (3) ≤ 50% sold, and (4) 0% sold, using maternal survey data on the livestock herd size and animals sold during the preceding year.

The study used the child fosterage variable also derived from survey data. Mothers listed all the children to whom they had given birth and indicated how many lived elsewhere. The variable children living at home was the number of children residing with the mother. The variable children fostered out was the number of children residing elsewhere. The survey data could not distinguish between fosterage and long‐term adoption, and ethnographically there is not a clear distinction between these practices among the study communities; therefore, fosterage and adoption were considered together. Because the raw variable children fostered out was right skewed and discontinuous, the statistical tests used the three‐level ordinal variable (none, one, and two or more). Since the interview did not ask why these children lived away from the mother, this variable does not differentiate fosterage/adoption from those away for other reasons. An alternative version of this variable was also derived by excluding older daughters who might have been married (> 15 years of estimated age).

Maternal nutritional deficiency variables were dichotomous, including underweight (BMI < 18.5 kg/m^2^), vitamin A deficiency (VAD; serum retinol < 1.05 μmol/L), and folate deficiency (serum homocysteine > 13 μmol/L; hyperhomocysteinemia). Hyperhomocysteinemia, a condition of elevated homocysteine (a sulfur amino acid) in blood, is a functional biomarker of deficiency in folate, vitamin B6, and/or B12; homocysteine accumulates in blood when a person has an inadequate level of any of these three B‐vitamins involved in the folate/methionine cycle (McKay and Mathers 2016). Human milk variables included energy (kcal/dl), retinol (μg/l), and folate receptor‐α (ng/ml). The latter is a folate‐binding protein that is highly correlated with milk folate and serves as a milk folate biomarker (Selhub et al. 1984; Tamura et al. 2009). The detailed description of how the maternal blood and milk samples were collected and analyzed can be found elsewhere (Fujita et al. 2019a, 2019b; Fujita et al. 2011; Fujita et al. 2022).

Adjustment variables included maternal age (years), parity, infant age (months), maternal inflammation (CRP > 5 mg/L; inflammation affects maternal energy budget and serum retinol levels), breastfeeding frequency, complementary feeding (yes or no), household size, and community (for further description of specimens/data collection/analysis, see Fujita et al. 2014; Vankayalapati et al. 2024).

### 2.3 Path Analysis

To evaluate the relationships in the hypothesized pathways linking behavioral variables, maternal nutritional status, and the milk variables, we applied the generalized structural equation model (GSEM) technique, which allows categorical and ordinal variables (Cain 2020). We did this in two steps. First, we estimated GSEM models for each of the dichotomous maternal nutritional variables as the response using the Bernoulli family with logit link, starting with the full set of predictors and adjustment variables. We used the Akaike Information Criterion (AIC) to assess the model fit. To strike the balance between parsimony and identifying potentially important associations in this exploratory study, the model selection strategy utilized AIC to identify the best‐fit model first and then log likelihood as an additional criterion to identify the optimal maternal nutrition model (Tredennick et al. 2021). Namely, of a subset of nested models having ∆AIC ≤ 2 of the lowest AIC value (i.e., a set of models largely indistinguishable as the best‐fit), an optimal model including one additional estimated parameter was selected if that parameter increased the log likelihood value compared to the best‐fit model (Leroux 2019). As part of model diagnostics, interactions between predictors of interest in the resulting models were checked. Only one interaction term was considered at a time.

Second, to each of the optimal maternal nutrition models, we added the maternal milk variable as the second response variable, including paths from the model's predictors of interest and maternal nutrition variable. From this full model, sequentially smaller nested models were estimated, guided by the same model selection strategy, except that a pathway from maternal nutrition to the milk response variable was retained in the model regardless of its effects. Interactions between predictors of interest were checked. Path diagrams were produced to depict key pathways in the final models, and summary tables were constructed for full details of final GSEM models.

For each apparent interaction between predictors of interest, a margins plot was constructed to visualize the nature of the interaction, using the predicted probability of maternal nutritional deficiency and expected values of milk nutrient content across the range of the hypothesized buffering variable.

Considering that hyperhomocysteinemia and VAD were relatively rare outcomes, we favored the power of Type II over Type I error (Kim and Choi 2021); we report regression models with 90% confidence intervals. Although the AIC method does not rely on the α‐level, the analysis used *p* < 0.1 to focus on the most pertinent associations.

## 3 Results

### 3.1 Sample Characteristics

Table 1 summarizes the sample characteristics. The mean ± SD maternal age was 28.16 ± 6.83 years, ranging from 18 to 46. The mean parity was 3.69 ± 2.24 (median 3), ranging from 1 to 12. The mean BMI was 19.78 ± 2.84, serum retinol was 1.50 ± 0.43 μmol/L and serum homocysteine was 9.15 μmol/L ± 3.24. Thirty‐three percent (72/221 mothers) of mothers were underweight, 13.2% (29/220 mothers) had VAD, and 10.8% (19/176 mothers) had hyperhomocysteinemia. The mean human milk energy, retinol, and folate receptor‐α concentrations were 72.27 ± 13.47 kcal, 27.71 ± 10.05 μg/L, and 610.8 ± 191.8 μg/mL, respectively. Milk energy and folate were comparable to the reported ranges from other populations (Miller et al. 2013; Selhub et al. 1982). Milk retinol was substantially lower than reported from other populations, almost at the lowest end of the reported human range (Fujita et al. 2017; Stoltzfus and Underwood 1995).

**TABLE 1:** Sample characteristics by socioeconomic status^a^ (*n* = 221).

	Overall (*n* = 221)				Low (*n* 57)		High (*n* 164)		*t‐*test *p*
	Mean	SD	Min	Max	Mean	SD	Mean	SD	
Maternal age (years)	28.16	6.83	18	46	27.44	6.39	28.41	6.97	0.35
Maternal parity	3.69	2.24	1	12	3.46	2.05	3.77	2.31	0.37
Maternal body mass index (kg/m^2^)	19.78	2.84	14.42	33.49	20.06	2.67	19.68	2.90	0.38
Serum retinol (μmol/l)^b^	1.50	0.43	0.40	3.05	1.57	0.37	1.48	0.45	0.19
Serum homocysteine (μmol/l)^c^	9.15	3.24	3.76	21.28	9.08	3.48	9.17	3.16	0.86
Milk energy (Kcal/dl)^d^	72.27	13.47	42.08	126.4	71.16	13.58	72.66	13.45	0.49
Milk retinol (μg/l)^e^	27.71	10.05	19	64	25.85	8.62	28.40	10.48	0.14
Milk folate receptor‐α (μg/ml)^f^	610.8	191.8	253.9	1248	622.2	185.4	606.7	194.4	0.62
Maternal serum C‐reactive protein	4.00	6.99	0.03	28.28^h^	4.09	7.22	3.96	6.93	0.91
Infant age (months)	8.02	4.50	0.80	19.50	7.81	4.84	8.09	4.39	0.68
Breastfeeding frequency	9.19	4.10	3	30	9.09	4.08	9.22	4.12	0.84
Children living at home	3.21	1.97	0	9	2.96	1.73	3.29	2.04	0.28
Children fostered out	0.31	0.91	0	9	0.35	0.67	0.29	0.98	0.68
Household size	5.32	2.09	2	12	4.91	1.82	5.46	2.17	0.09
Land size (acres)	2.33	3.45	0	26	0.18	0.38	3.08	3.72	0.00
TLU^g^ per household	1.66	4.52	0	32	0.22	0.33	2.17	5.15	0.00
Cattle held per household	1.05	3.37	0	40	0.14	0.35	1.36	3.86	0.01
Goats/sheep held per household	4.30	12.87	0	100	1.04	2.09	5.44	14.73	0.03

^a^ Socioeconomic status based on the composite measure of self‐rated poverty (y/n), livestock holding, and land size.

^b^ *n* 220 overall, *n* 57 low SES, *n* 163 high SES.

^c^ *n* 176 overall, *n* 49 low SES, *n* 127 high SES.

^d^ *n* 202 overall, *n* 53 low SES, *n* 149 high SES.

^e^ *n* 173 overall, *n* 47 low SES, *n* 126 high SES.

^f^ *n* 202 overall, *n* 53 low SES, *n* 149 high SES.

^g^ Tropical livestock unit: 1.0 camel, 0.8 cattle, 0.10 small stock (goats and sheep).

^h^ Assay upper limit of detection.

The mean ± SD infant age was 8.02 ± 4.50 months, ranging from 0.8 to 19.50. All infants were breastfed (by study design) with the mean nursing frequency of 9.19 ± 4.1, ranging from 3 to 30 times over 24 h. Seventy‐nine infants (36%) were exclusively breastfed. The mean number of children living at home was 3.21 ± 1.97 with the median of three. The mean number of children fostered out was 0.31 ± 0.91 with the median of zero. Forty out of 221 households (18.10%) fostered out one or more children. The mean household size was 5.32 ± 2.09, ranging from 2 to 12. The mean land size was 2.33 ± 3.45 acres. TLU at the time of the survey (i.e., the balance after livestock sales) was 1.66 ± 4.52. At the time of the survey, the mean number of cattle held was 1.05 (range 0–40) and the mean number of goats/sheep held was 4.30 (range 0–100). Sixty‐five percent of the households had no cattle remaining at the time of the survey while 51% had no goats/sheep remaining. Households with large herds constituted a small minority: only 5% of households held five or more cows while 10% of households held 10 or more goats/sheep.

When mothers were classified dichotomously using the composite socioeconomic scale that drew on both the emic (maternal self‐rating of poverty) and etic (land size and TLU) criteria for descriptive purposes (for details see (Fujita et al. 2012a)), the maternal and children's residency characteristics did not differ noticeably (Table 1). However, household size was slightly larger in high SES (5.46 vs. 4.91, *P* = 0.09) and maternal underweight was more prevalent in high SES (36.6% vs. 21.1% in low SES, Pearson χ^2^_(1)_ = 4.65, *P* = 0.031).

Table 2 shows the frequency of households with livestock sold/children fostered out, stratified by SES. There was an association between cattle sold and SES (Pearson χ^2^_(3)_ = 15.380, *P* = 0.002). Most notably, low SES had more households with no cattle at the start of the year (75.4%) than high SES (46.3%). There was a similar pattern for goats/sheep sold. Conversely, low SES households tended to have proportionally more children fostered out (26.3% at least one child fostered out) than high SES households (15.2%; Pearson χ^2^_(2)_ = 3.954, *P* = 0.138). The correlation between the number of children fostered out and children living at home was negligible (Pearson's *r* = −0.087, *P* = 0.198).

**TABLE 2:** Frequency of households with livestock sales/children fostered out by socioeconomic status.

	Socioeconomic status		Total
	Low	High	
A. Cattle sold			
Had no cattle	43 (75.4%)	76 (46.3%)	119 (54%)
> 50% cattle sold	6 (10.5%)	27 (16.5%)	33 (15%)
≤ 50% cattle sold	1 (1.8%)	17 (10.4%)	18 (8%)
No cattle sold	7 (12.3%)	44 (26.8%)	51 (23%)
Total	57 (100.0%)	164 (100.0%)	221 (100.0%)
B. Goats/sheep sold			
Had no goats/sheep	29 (50.9%)	66 (40.2%)	95 (43.0%)
> 50% goats/sheep sold	8 (14.0%)	15 (9.1%)	23 (10.4%)
≤ 50% goats/sheep sold	8 (14.0%)	26 (15.9%)	34 (15.4%)
No goats/sheep sold	12 (21.1%)	57 (34.8%)	69 (31.2%)
Total	57 (100.0%)	164 (100.0%)	221 (100.0%)
C. Children fostered out			
None	42 (73.7%)	139 (84.8%)	181 (81.9%)
One	11 (19.3%)	16 (9.7%)	27 (12.2%)
Two or more	4 (7.0%)	9 (5.5%)	13 (5.9%)
Total	57 (100.0%)	164 (100.0%)	221 (100.0%)

*Note:* A. Pearson χ^2^_(3)_ = 15.380, *P* = 0.002; B. Pearson χ^2^_(3)_ = 4.719, *P* = 0.194; C. Pearson χ^2^_(2)_ = 3.954, *P* = 0.138.

### 3.2 Bivariate Associations Between Behavioral Buffering Variables

**FIGURE 2:**
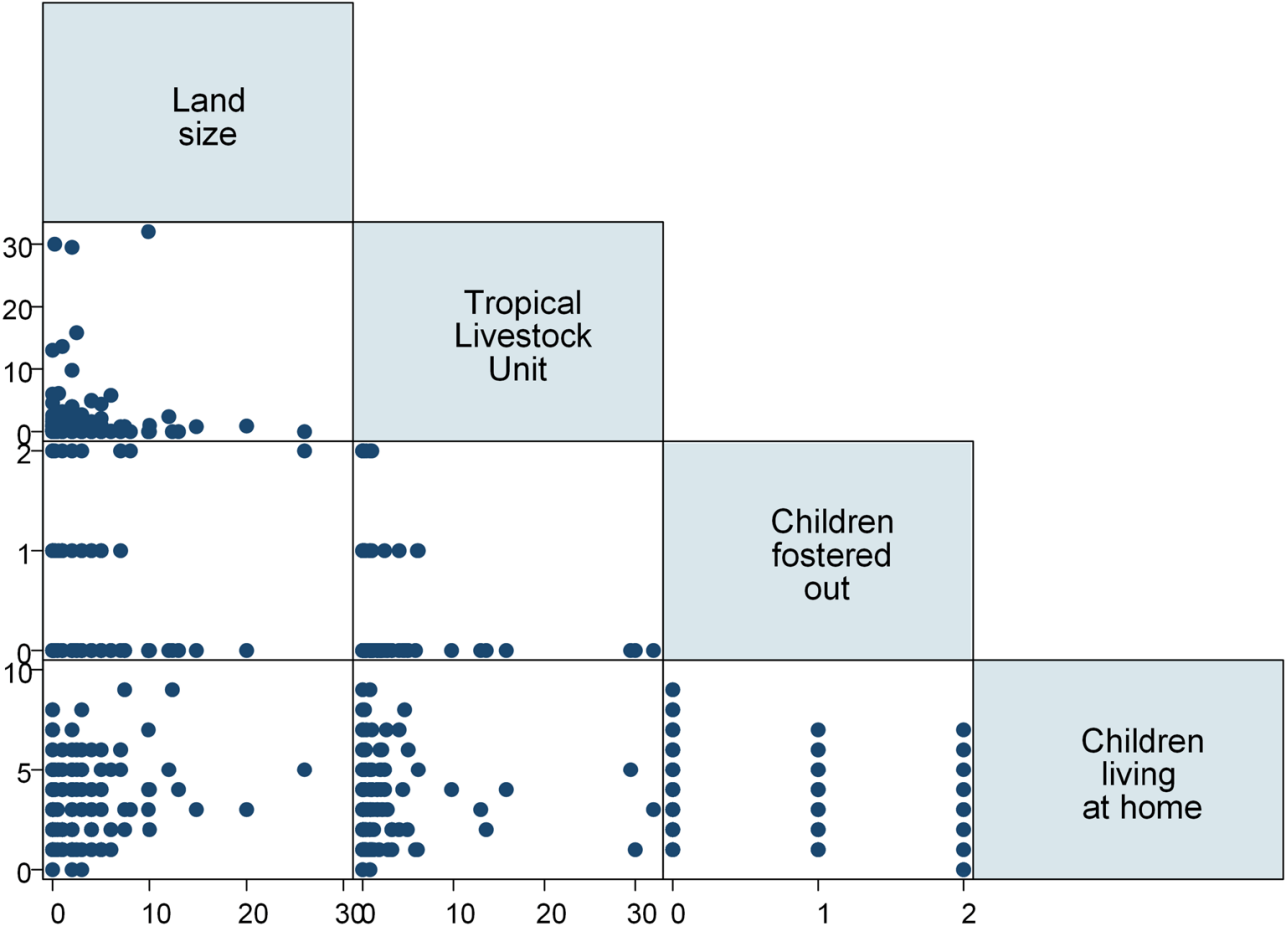
Scatterplot matrix of economic/behavioral variables. More children were living at home in households with greater land size (Pearson's *r* = 0.15, *P* = 0.021). More children were fostered out in households with smaller livestock holding (Spearman's rho = −0.11, *P* = 0.097). TLU, Tropical Livestock Unit.

Scatter plots for economic/behavioral buffering variables—land size, TLU, children fostered out, and children living at home—are shown in Figure 2. There was a positive correlation between land size and children living at home (Pearson's *r* = 0.15, *P* = 0.021); more children lived at home as the land size increased. There was an inverse correlation between TLU and children fostered out (Spearman's rho = −0.11, *P* = 0.097); more children were fostered out in households with smaller TLU. Other pairwise correlations were not apparent (*r* or *rho* ≤ 0.1 and *p* > 0.1).

To allow for livestock species‐specific statistics, we further examined pairwise associations between children fostered out (y/n) and livestock held (y/n), differentiating cattle and goats/sheep (Table 3). The proportion of child fosterage was greater among households with no cattle held (either had none to sell or sold all animals) than households with some cattle held (22.8% vs. 9.2%; Pearson χ^2^_(1)_ = 6.17, *P* = 0.013). The pattern was the same for goats/sheep held, albeit with less apparent contrast (20.4% vs. 15.9%; χ^2^_(1)_ = 0.735, *P* = 0.391).

**TABLE 3:** Household cattle/goats/sheep holding by children fostered out (y/n).

	Children fostered out		Total
	No	Yes	
A. Cattle held			
No	112 (77.2%)	33 (22.8%)	145 (100.0%)
Yes	69 (90.8%)	7 (9.2%)	76 (100.0%)
Total	181 (81.9%)	40 (18.1%)	221 (100%)
B. Goats held			
No	86 (79.6%)	22 (20.4%)	108 (100.0%)
Yes	95 (84.0%)	18 (15.9%)	113 (100.0%)
Total	181 (81.9%)	40 (18.1%)	221 (100%)

*Note:* A. Pearson χ^2^_(1)_ = 6.17, *P* = 0.013; B. Pearson χ^2^_(1)_ = 0.735, *P* = 0.391.

### 3.3 Path Analysis in GSEM Models

The summary of the selection process (AIC, ∆AIC, etc.) for the paths to maternal nutrition responses can be found in (Table S1). The optimal models for maternal nutrition included land size, children fostered out and/or children at home, and cattle or goat sold. The final GSEM models including relevant pathways from behavioral buffering variables to the maternal nutrition and milk responses are summarized in Table S2 panels a, b, and c for underweight‐milk energy, VAD–milk retinol, and hyperhomocysteinemia‐milk folate, respectively.

#### 3.3.1 Maternal Underweight and Maternal Milk Energy

**FIGURE 3:**
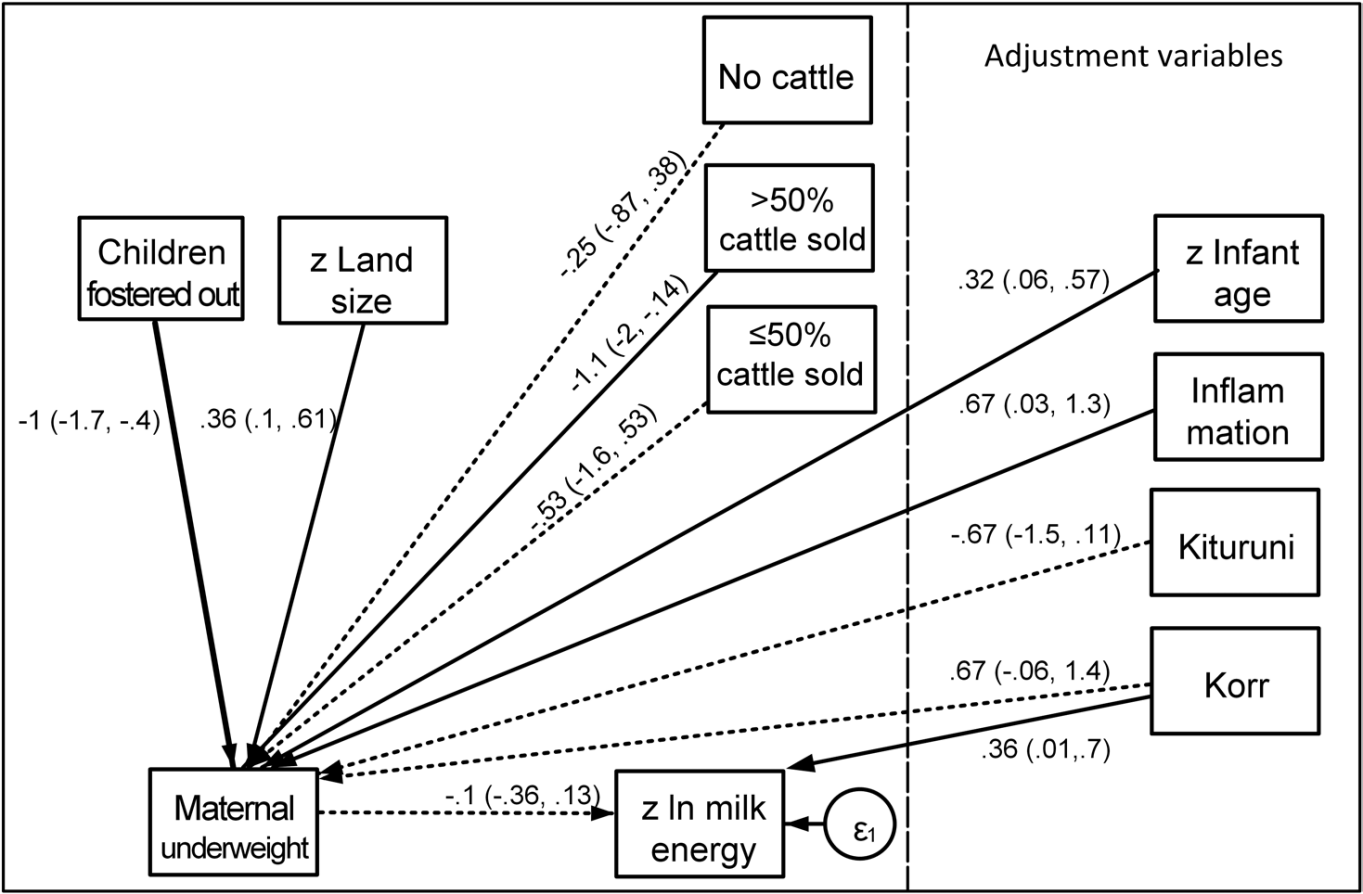
Path diagram for the effects of buffering variables on maternal underweight and milk energy. Thick arrow *p* < 0.01, solid arrow *p* < 0.1, dashed arrow *p* ≥ 0.1; shown with path coefficients with 90% CI.

Figure 3 presents the final GSEM model for maternal underweight and milk energy. Among the hypothesized pathways connecting predictors of interest and maternal underweight, maternal underweight was inversely associated with children fostered out (Coef. = −1.05, 90% CI = −1.70, −0.40; *P* = 0.008) and positively associated with land size (Coef. = 0.36, 90% CI = 0.10, 0.61; *P* = 0.021) (Table S2a), adjusted for infant age, inflammation, and community. Cattle sold, particularly > 50% sold, was inversely associated with underweight (Coef. = −1.08, 90% CI = −2.01, −0.14; *P* = 0.059). There were no interactions between the predictors of interest. There were no significant pathways linking the predictors of interest or maternal underweight to milk energy.

#### 3.3.2 Maternal Vitamin A Deficiency and Maternal Milk Retinol

**FIGURE 4:**
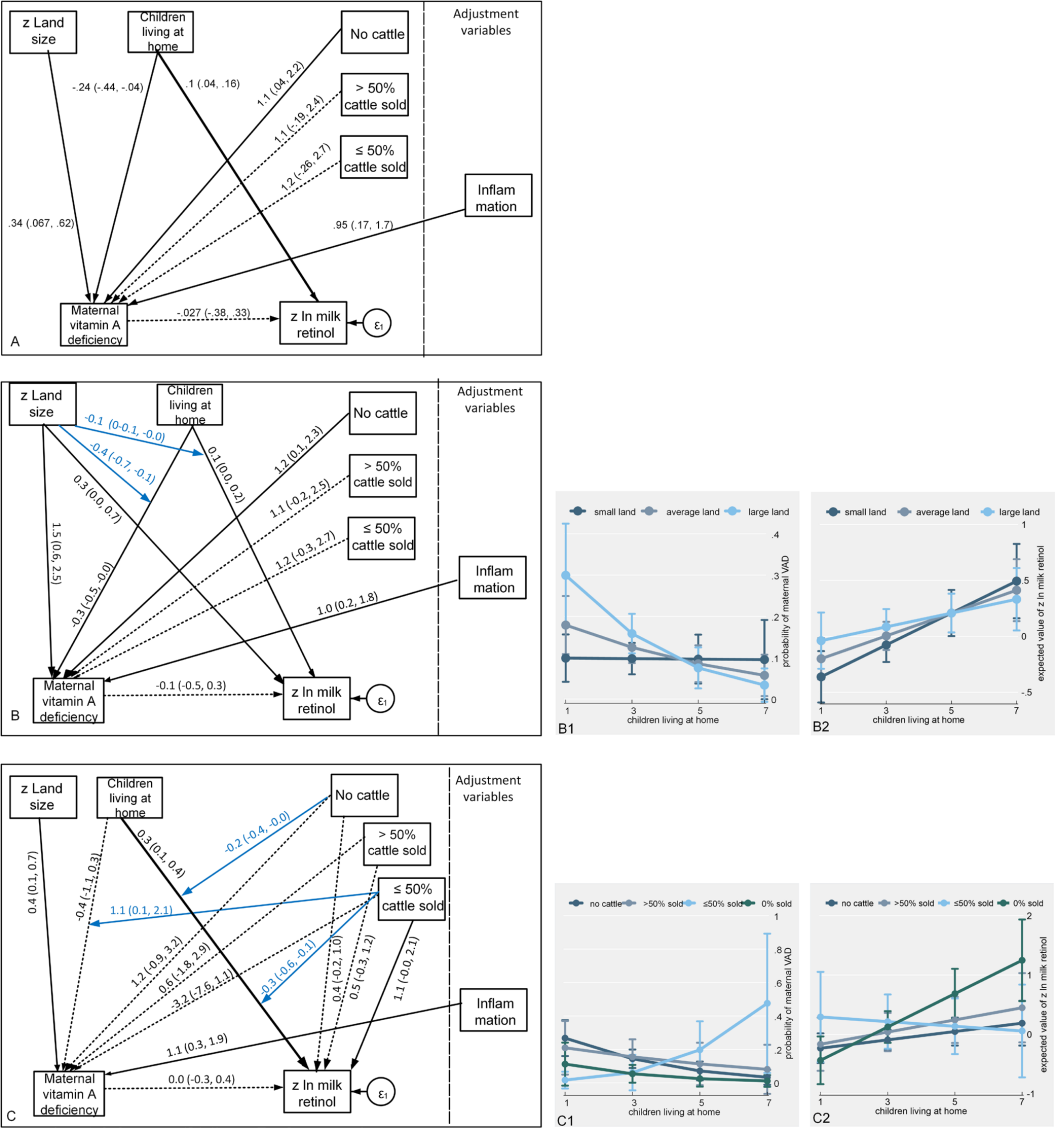
Path diagrams for the effects of buffering variables on maternal vitamin A deficiency and milk retinol: (a) main effects only, (b) with children–land interaction, and (c) with children–cattle interaction, with predictive margins plots. Thick arrow *p* < 0.01, solid arrow *p* < 0.1, dashed arrow *p* ≥ 0.1, shown with path coefficient with 90% CI. Blue arrows show moderating effects.

Figure 4 presents the final GSEM models for maternal VAD and milk retinol. In the main‐effect model (Figure 4 panel a; Table S2b, Model A), maternal VAD was inversely associated with children living at home (Coef. = −0.24, 90% CI = −0.44, −0.04; *P* = 0.054) and positively associated with land size (Coef. = 0.34, 90% CI = 0.07, 0.62; *P* = 0.041), adjusted for maternal inflammation. Maternal VAD was positively associated with having no animals to sell (Coef. = 1.13, 90% CI = 0.04, 2.22; *P* = 0.089) compared to the reference category none sold. There were interactions between children living at home and land size, and between children living at home and cattle sold.

Accounting for the interaction between children living at home and land size (Coef. = −0.36, 90% CI = −0.7, −0.05; *P* = 0.052; Figure 4 panel b; Table S2b Model B) resulted in a stronger coefficient for the path linking land size and VAD (Coef. = 1.54, 90% CI = 0.58, 2.51; *P* = 0.009) and minimal change in other paths. This interaction suggests that the protective effect of children living at home was limited to above‐average land size (Figure 4b plot 1). Namely, for mothers with large land (at the 85th percentile), the predicted probability of VAD with one child living at home was 0.30 (90% CI = 0.17, 0.42) but only 0.03 (90% CI = −0.01, 0.01) with seven children living at home. In contrast, among mothers with small land (at the 15th percentile), the predicted probability of VAD did not change with children living at home. Likewise, children living at home were positively associated with milk retinol (Coef. = 0.10, 90% CI = 0.04, 0.16; *P* = 0.007) in the main‐effect model, but there was also an interaction between children living at home and land size (Coef. = −0.07, 90% CI = −0.13, −0.001; *P* = 0.094). This interaction suggests that the positive effect of children living at home was stronger for smaller land size, as shown in the margins plot (Figure 4b plot 2) with a steeper rise in the expected milk retinol content across the range of children living at home among mothers with small land (the 15th percentile) than those with larger land.

The model accounting for the interaction between children living at home and cattle sold (particularly ≤ 50% sold; Coef. = 1.13, 90% CI = 0.13, 2.13; *P* = 0.06) is shown in Figure 4 panel c (Table S2b Model C). This interaction modified the effect of children living at home on both VAD and milk retinol. Namely, while the variable children living at home was generally inversely associated with VAD, this was not the case for the category ≤ 50% cattle sold; the predicted probability of maternal VAD increased as children living at home increased only in households that sold ≤ 50% of the cattle herds (Figure 4c plot 1). Meanwhile, for milk retinol, this interaction (Coef. = 1.13, 90% CI = 0.13, 2.13, *P* = 0.064 for ≤ 50% sold) suggests that although children living at home were generally positively associated with milk retinol (Coef. = 0.28, 90% CI = 0.13, 0.43, *P* = 0.002), it was unassociated with milk retinol in the category of households that sold ≤ 50% of the cattle herds.

#### 3.3.3 Maternal Hyperhomocysteinemia and Maternal Milk Folate Receptor‐α

**FIGURE 5:**
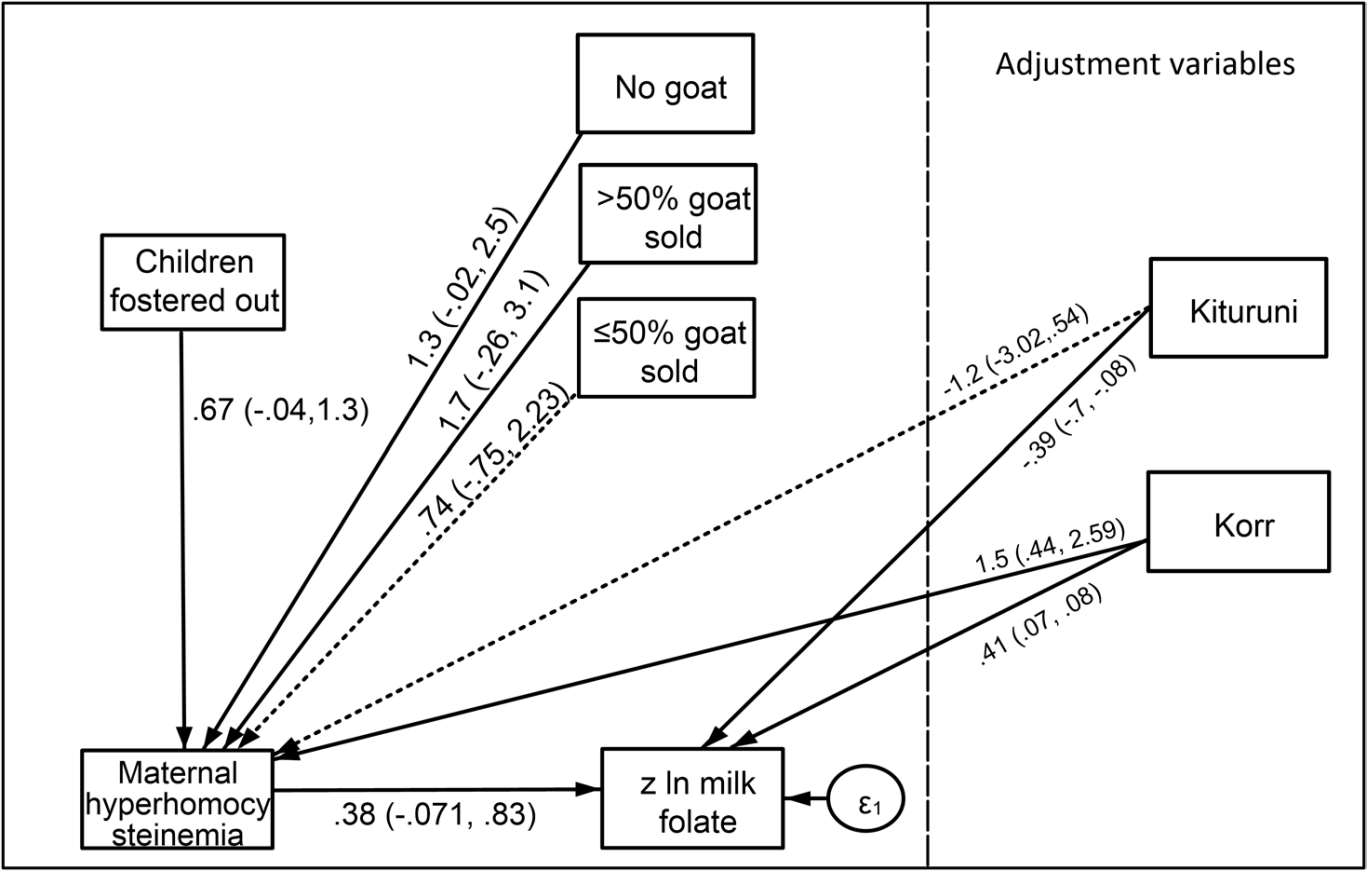
Path diagram and predictive margins graphs for the effects of buffering variables on hyperhomocysteinemia and milk folate receptor‐α. Solid arrow *p* < 0.1, dashed arrow *p* ≥ 0.1.

Maternal hyperhomocysteinemia was positively associated with children fostered out (Coef. = 0.67, 90% CI = 0.04, 1.30; *P* = 0.08; Figure 5, Table S2C), adjusted for community. Goats/sheep sold, particularly having sold > 50% of goats/sheep (Coef. = 1.69, 90% CI = 0.26, 3.13; *P* = 0.052) and having no goats/sheep to sell (Coef. = 1.27, 90% CI = 0.02, 2.52; *P* = 0.095), were positively associated with hyperhomocysteinemia.

Maternal underweight‐milk energy and maternal hyperhomocysteinemia‐milk folate models estimated using the alternative child fosterage variable excluding older daughters did not noticeably alter the models (Table S3).

## 4 Discussion

Table 4 summarizes the overall results of GSEM models and interpretations of path coefficients. Maternal nutritional deficiency outcomes were often adversely associated with economic and other behavioral buffering variables, while any of the milk nutrient content variables investigated here were not adversely associated with these predictor variables. This contrast suggests that the behavioral buffering examined here can offer some protection against maternal malnutrition and that maternal physiological buffering of milk content closes much of the gap left by incomplete behavioral buffering.

**TABLE 4:** Summary of behavioral buffering path coefficient interpretations.

Behavioral buffering variable	Energy		Retinol		Folate	
	Maternal underweight	Milk energy	Maternal vitamin A deficiency	Milk retinol	Maternal folate deficiency	Milk folate
Land size owned	Adverse	No effect	Adverse	Beneficial	No effect^a^	Not evaluated^a^
	Milk is physiologically buffered		Milk is physiologically buffered		Maternal nutrition is buffered	
Children fostered out	Beneficial	No effect	No association^a^	Not evaluated^a^	Adverse	No effect
	Maternal nutrition is buffered		Maternal nutrition is buffered		Milk is physiologically buffered	
Children living at home	No effect^a^	Not evaluated^a^	Beneficial^b^	Beneficial^b^	No effect^a^	Not evaluated^a^
	Maternal nutrition is buffered		Maternal nutrition is buffered		Maternal nutrition is buffered	
Livestock sold	Beneficial with > 50% cattle sold	No effect	Adverse with no cattle to sell and ≤ 50% cattle sold^b^	No effect or beneficial with ≤ 50% cattle sold^c^	Adverse with no goats/sheep to sell or > 50% goats/sheep sold	No effect
	Maternal nutrition is buffered		Milk is physiologically buffered		Milk is physiologically buffered	

^a^ Path to maternal nutrition response dropped in model selection; thus, the path to milk response variable was not evaluated.

^b^ Depending on land size or cattle sold.

^c^ Depending on children at home.

### 4.1 Land Size

Land size was a robust predictor of maternal malnutrition. Agricultural economic activities may leave breastfeeding women more vulnerable to drought. Women whose households were more invested in agriculture, as captured by land size, were more likely to have underweight and VAD. This is consistent with the government report (Marsabit County Government 2018) identifying those who rely on cultivation as the most vulnerable to the adverse impacts of extreme weather associated with climate change.

In contrast, land size was unassociated with milk energy and positively associated with retinol. This suggests that maternal physiological buffering shielded milk energy and retinol content against mothers' own nutritional deficiency that may be linked to investment in land.

### 4.2 Children Fostered Out

Another robust predictor of maternal malnutrition was children fostered out, suggesting that fostering or adopting children out of the household could help buffer some aspects of maternal nutrition, but not others: namely, mothers with more children fostered out were less likely to be underweight but more likely to have hyperhomocysteinemia and therefore folate or other B vitamin deficiency. This suggests that fostering out children could be an effective buffering strategy to protect mothers against underweight (likely arising from overall shortage of food or calories), but that this strategy was likely incomplete in mitigating food stress, as hyperhomocysteinemia likely arises from poor quality of the diet, rather than a shortage of food or calories. In bivariate analysis, mothers in the worst circumstances, such as those with no livestock, were more likely to have one or more children fostered out. Together, these results suggest that mothers who fostered children to other households were successful in alleviating nutritional strains around the quantity of food or calories they could access for their households to a greater extent than strains around the quality of those foods.

In contrast, neither milk energy nor milk folate was associated with children fostered out. This suggests that child fosterage offered effective buffering for both maternal protein‐energy status and milk energy, but less effective buffering for maternal folate and milk folate. Indeed, fostering children out was associated with higher (rather than lower) rates of maternal folate deficiency, suggesting that this behavioral buffer did not protect maternal folate nutrition; lactation physiology seems to have closed the gap through physiological buffering of milk folate content. This is consistent with our previous study that found maternal folate delivery to milk is highest for a subset of mothers with hyperhomocysteinemia (Fujita et al. 2022).

Child fosterage is common in much of sub‐Saharan Africa; some estimate that over 50% of adults were fostered out of their natal homes at one point in their lifetime (Archambault and de Laat 2010). Multiple sources describe child fostering in sub‐Saharan African context as a risk mitigation strategy that can redistribute parenting burden across households, which can promote the wellbeing, educational success, and survival of fostered children (Archambault and de Laat 2010). This is one expression of their ethos of communal parenting which parallels communal herding. Among Kenyan pastoralists, families cooperate to carry out domestic tasks including childcare; for example, among Maasai herders' homesteads, each mother commonly lives with her biological children as well as children of others, and children move between houses, performing “chores for other mothers or eating and playing in other houses” (Archambault and de Laat 2010; Talle 2004). Additionally, child adoption within the patrilineal or extended families often occurs to equalize wealth (including children) from parous mothers to childless woman to achieve balance and moral order (Talle 2004).

We add to this literature by demonstrating that child fostering among pastoralists and agropastoralists in northern Kenya may be a nutritional risk mitigation strategy, particularly for protein‐energy nutrition of some of the most vulnerable: breastfeeding mothers and nursing infants. This further corroborates the notion of child fosterage as a reproductive strategy (Pennington 1991) and cooperative breeding (Prall and Scelza 2017; Scelza and Silk 2014) in an evolutionary understanding of nutritional and/or reproductive benefits of child fosterage in African contexts. However, our findings provide cautionary evidence that child fostering can entail covert yet high maternal costs, as maternal physiology fills the nutritional gap left by the incomplete behavioral buffering of maternal folate. This high cost, given the fundamental roles that folates play in biological processes underlying reproduction, might reduce the reproductive benefit of child fosterage's buffering of maternal and milk energy and may complicate the evolutionary understanding of child fosterage as a reproductive strategy.

### 4.3 Children Living at Home

Number of children living at home was a protective/beneficial factor in models of both maternal VAD and milk retinol; however, the effect on VAD and milk retinol varied depending on both land size and cattle sold. Namely, having more children living at home was associated with decreased probability of VAD; this protective effect was stronger for households with more land. Meanwhile, having more children living at home was beneficial for milk retinol content, particularly in those with small land. Similarly, the protective effect of children at home on VAD was less for households with fewer cattle sold, while the beneficial effect of children at home on milk retinol was attenuated in those households. We can offer a few likely explanations for these patterns, all related to the contributions of these children to household activities: Children living at home may have provided infant allocare (Meehan et al. 2013; Vankayalapati et al. 2024; Waynforth 2020), allowing mothers to pursue activities that generated high quality, vitamin A‐rich food for themselves and their families.

Children living at home may also have shifted labor needs and allocation within their households. Among neighboring Samburu pastoralists newly adopting cultivation in the 2020s, labor burdens shifted to women, particularly when there was insufficient child labor at home, most often for young wives (Straight, personal observation). A time allocation study (Straight 1997) also chronicled an increased labor burden for women when children's labor was lost to education. Conversely, in a lowland setting with large herds, the labor burden on young wives could be alleviated by daughters, who could take on herding obligations to free their brothers to attend school (Iannotti et al. 2022; Straight et al. 2025). Therefore, having more children at home can increase the opportunity for mothers to delegate strenuous household tasks (including caring for livestock or tending farms), providing relief for maternal physical labor and therefore nutritional burden. However, such relief, whether through children's allocare or contribution to household economy, likely depends on the balance between the number of children old enough to positively contribute and the economic capital (livestock or land). The shift in this balance may be captured by the interaction we observed between land size and children living at home.

It should be noted that since milk retinol concentrations were overall substantially lower than those reported from other populations (Fujita et al. 2017; Stoltzfus and Underwood 1995), effective buffering here does not translate to adequate milk retinol content. Rather, it means that having more children predicted higher milk retinol than others within the sample.

### 4.4 Livestock Sold

#### 4.4.1 Cattle Sold

The results from GSEM models showed the possibility that livestock sales increased or decreased the risk of maternal malnutrition, depending on what aspect of nutrition was considered, what proportions of which livestock species were sold, and for maternal VAD/milk retinol, how many children lived at home. Selling cattle, particularly selling a substantial proportion of the cattle herd, was protective against maternal risk for underweight, and selling cattle was unassociated with milk energy. This suggests that cattle sales provided an effective behavioral buffer for maternal protein‐energy nutrition and milk energy.

Yet, we also found that selling cattle, particularly selling fewer (≤ 50%) of them, was positively associated with maternal VAD, particularly as the number of children living at home increased. Selling ≤ 50% of the cattle herd also attenuated the positive effect of children living at home on milk retinol. These results suggest that while selling cattle could be an effective behavioral buffering for maternal and milk vitamin A, it was less effective when fewer cattle were sold and in households with more children.

These data were collected after multiple years of poor rainfall, which depleted herds such that very few of our participants had large herds; thus “selling > 50%” typically represents families selling one or more of their last remaining animals, particularly for cattle. Liquidating cattle in the context of a drought seems to have saved mothers from food shortfall (as reflected by protection against underweight) but did not protect overall diet quality (as evidenced by increasing risk for VAD).

It is unclear what underlies the interaction between cattle sold, particularly fewer cattle sold, and children living at home. It may be attributable to women's dual responsibility to care for children and cattle of the household, combined with the heightened scarcity and rising market prices of vitamin A‐rich foods during drought. In northern Kenyan pastoralists, women work long hours, with a large proportion of time devoted to caring for children, daily milking of livestock (cattle and goats/sheep), providing veterinary care for nursing stock, and purchasing foods for household consumption, which may be financed by women selling livestock products such as surplus cattle milk (Fratkin and Smith 1995). Mothers with more children at home may have faced higher labor demands than those with fewer dependent children and fewer cattle (although this difference may be subtle, as neighboring households often cooperate in care of both children and livestock). Vitamin A‐rich foods (such as milk, eggs, and dark‐green leafy vegetables) typically become scarcer and more expensive in prolonged drought, and cattle milk yields diminish (McCabe 1987). Mothers may opt for more affordable, low‐quality foods to try to make them last, and likely devote their diminished cattle milk to feeding children or for sale. This can decrease household dietary quality overall, with the strongest decrease in maternal dietary quality. Agricultural economists have reported that adverse nutritional impacts of food insecurity are most apparent in individuals who make household food allocation decisions—mothers among the Ariaal (Villa et al. 2011). We posit that the interaction between cattle sold and children at home suggests that mothers of households that sold fewer cattle had progressively diminished retinol somatic reserves with an increasing number of children at home because they preferentially allocated quality food to children.

#### 4.4.2 Goats/Sheep Sold

Goats/sheep sold were positively associated with maternal hyperhomocysteinemia, a condition indicative of folate deficiency, further suggesting that selling livestock can offer only incomplete buffering for maternal micronutrient nutrition in this context. Maternal risk for hyperhomocysteinemia was highest for those who sold the majority of their goats/sheep and those who had no goats/sheep to sell. This suggests that selling some goats/sheep can buffer maternal folate nutrition when a large proportion of the herd is kept, perhaps reflecting the importance of goat‐source foods for mothers' folate nutrition, particularly during breastfeeding, when folate requirements are elevated (Tamura et al. 2009). Goats tend to have higher tolerance than cattle for prolonged droughts (McCabe 1987), and goat milk (or flesh) may be a superior source of essential nutrients to purchased or drought‐relief distributed foods (Turkmen 2017). In northern Kenyan pastoralists, goats (and sheep) are typically more readily sold than cattle (Fratkin and Roth 1990). Having to sell the majority or all of goats/sheep implies a dire situation for the household economy. Selling the goats/sheep may allow households to buy enough foods for the household, but at the expense of highly nutritious goat‐derived foods.

Selling goats/sheep was not associated with maternal milk folate content. Furthermore, maternal hyperhomocysteinemia predicted higher milk folate content. The lack of adverse effects of goats/sheep sold on human milk folate content despite the adverse effects on maternal folate nutrition, as well as the higher milk folate content in the presence of maternal hyperhomocysteinemia, suggests physiological buffering closed the gap left by incomplete behavioral buffering even at the expense of mothers' own nutrition.

## 5 Limitations

This study drew on available data from a previously conducted survey, which was not collected specifically for these analyses. Our variable children fostered out utilized information about children living away from participating mothers as a surrogate measure for child fosterage. The above‐reported associations between children fostered out and maternal nutritional deficiency therefore may be in part attributable to children studying/working elsewhere, or, for older daughters, being married. Research among the Maasai has found that fosterage is most commonly motivated by educational access (Archambault and de Laat 2010). The decision to foster out children for education may or may not have a direct relationship with coping with drought‐related stress. However, our interest lies in the dynamic distribution of children across households as an economic/behavioral strategy, and not in the specific reasons for their absence from a household. Marriage may be qualitatively different in that it reflects a more permanent life stage transition than a coping behavior; however, removing daughters of potentially marriageable age from our definition of children fostered out did not alter our findings. Another common reason for child fosterage among the Maasai was to assist a relative with household duties such as livestock herding, often with larger middle‐aged families fostering out children to aid smaller young families or the elderly (Archambault and de Laat 2010). The results from the present study appear consistent with this kind of movement of children across households.

We relied on data from a cross‐sectional survey. As such, it is not possible to assign a causal direction to the associations we observed. For example, mothers with poorer nutrition might have sold livestock at different rates, rather than selling livestock altering their diets to impact their nutrition. We nonetheless provide these possibilities for future research to investigate with a prospective research design.

Finally, there is a need to be cautious with the concept of “buffering”. We defined the lack of an inverse association between behavioral variables or maternal nutrition and human milk content as evidence for buffering. However, milk nutrient concentration does not perfectly capture the amount of energy or micronutrient delivered to infants via milk, which is also affected by the total volume of milk an infant consumes. We did not collect information on the milk volume consumed by infants, and so we cannot be certain that support for our predictions around “buffering” can be interpreted as adequate energy/nutrient delivery to infants via milk. The infant feeding/supplementation strategy could be another behavioral buffer that might not be captured in our models. However, we did include complementary feeding and breastfeeding frequency in the list of initial variables for model selection. Neither variable was associated with maternal nutritional outcomes. Additionally, the retinol concentrations in milk we observed were extremely low overall. While we found support for physiological buffering, we cannot conclude that participating infants were at particularly low risk for VAD.

## 6 Conclusion

This study explored how economic and other behavioral coping strategies pastoralists and agropastoralists employ during droughts might buffer maternal and human milk nutrition, focusing on three different aspects of nutrition that are of fundamental importance for human reproduction: energy, vitamin A, and folate. We found that agropastoralists' practice of building livestock herds whenever possible and downsizing during severe droughts to make ends meet, as well as using child fosterage to manage household labor and food needs, depending on the economic and ecological situations, are behavioral strategies that can partially buffer the nutritional status of breastfeeding mothers. These findings highlight the importance of investment in a sustainable livestock herd economy for the health of agropastoralists.

The study also contextualized the capacity of human lactation physiology to buffer milk, and therefore infants, against maternal nutritional stress, closing the gap left by the behavioral buffering. Further, physiological buffering of milk may come at a heavy nutritional cost for breastfeeding mothers during the severe stress of a drought, particularly for their micronutrient health. Given the increasing severity and frequency of droughts, nutritional burdens on mothers may be increasing. Support for reproductive‐aged women should be prioritized in public health policy decisions concerned with pastoralists in East Africa and other arid regions of the world.

## Conflicts of Interest

The authors declare no conflicts of interest.

## Supporting information


Data S1.

## Data Availability

The data that support the findings of this study are openly available in Zenodo at https://zenodo.org/records/13285825.
