# Supplementary material for: Dual Behavioral–Physiological Buffering of Mothers' Milk Facilitates Drought Adaptability of Pastoralists and Agropastoralists in Northern Kenya
Source: Am J Hum Biol. 2025 May 19;37(5):e70057. doi: 10.1002/ajhb.70057 (PMC12086614; doi:10.1002/ajhb.70057)
Supplement: Supplementary file 1 — Data S1. [file AJHB-37-e70057-s001.docx]

Supplementary Information

## Table S1 Model selection for maternal nutritional status

| **A. Outcome: Maternal underweight ^a^** | | | | | | | | | | | | | |
| --- | --- | --- | --- | --- | --- | --- | --- | --- | --- | --- | --- | --- | --- |
|  | **Model 1** | **Model 2** | **Model 3** | **Model 4** | **Model 5** | **Model 6** | **Model 7** | **Model 8** | **Model 9** | **Model 10** | **Model 11** | **Model 12** | **Model 13** |
|  |  |  |  |  |  |  |  | **Optimal model** |  | **Lowest AIC** |  |  |  |
|  | Cattle sold | Cattle sold | Cattle sold | Cattle sold | Cattle sold | Cattle sold | Cattle sold | Cattle sold | Cattle sold | x | x | x | x |
|  | Goat sold | x | x | x | x | x | x | x | x | x | x | x | x |
|  | Children fostered out | Children fostered out | Children fostered out | Children fostered out | Children fostered out | Children fostered out | Children fostered out | Children fostered out | Children fostered out | Children fostered out | Children fostered out | Children fostered out | Children fostered out |
|  | Children living at home | Children living at home | Children living at home | Children living at home | Children living at home | Children living at home | Children living at home | x | x | x | x | x | x |
|  | Land size | Land size | Land size | Land size | Land size | Land size | Land size | Land size | Land size | Land size | Land size | Land size | Land size |
|  | Maternal age | Maternal age | Maternal age | Maternal age | Maternal age | Maternal age | x | x | x | x | x | x | x |
|  | Infant age | Infant age | Infant age | Infant age | Infant age | Infant age | Infant age | Infant age | Infant age | Infant age | Infant age | x | x |
|  | Community | Community | Community | Community | Community | Community | Community | Community | x | Community | Community | Community | x |
|  | Inflammation | Inflammation | Inflammation | Inflammation | Inflammation | Inflammation | Inflammation | Inflammation | Inflammation | Inflammation | x | Inflammation | Inflammation |
|  | Parity | Parity | Parity | x | x | x | x | x | x | x |  |  |  |
|  | Household size | Household size | Household size | Household size | x | x | x | x | x | x |  |  |  |
|  | Complementary feeding | Complementary feeding | Complementary feeding | Complementary feeding | Complementary feeding | x | x | x | x | x |  |  |  |
|  | Breast feeding freq. | Breast feeding freq. | x | **x** | x | x | x | x | x | x |  |  |  |
| **AIC** | 284.9 | 280.0 | 278.2 | 276.3 | 274.7 | 273.3 | 272.8 | 271.8 | 272.7 | 270.0 | 271.4 | 273.2 | 273.5 |
| **∆AIC** | 15.0 | 10.1 | 8.2 | 6.4 | 4.7 | 3.3 | 2.8 | 1.8 | 2.7 | 0.0 | 1.4 | 3.2 | 0.8 |
| **BIC** | 349.4 | 334.3 | 329.1 | 323.8 | 318.8 | 314.0 | 310.1 | 305.7 | 299.8 | 293.7 | 291.7 | 290.2 | 283.7 |
| **LL** | -123.5 | -124.0 | -124.1 | -124.2 | -124.3 | -124.6 | -125.4 | -125.9 | -128.3 | -128.0 | -129.7 | -131.6 | -133.7 |
| **n** | 220 | 220 | 220 | 220 | 220 | 220 | 220 | 220 | 220 | 220 | 220 | 220 | 220 |

^a^ Model selection was done restricting the sample size to n 220 for fair comparisons of AIC values across nested models. The selected models were then rerun for n 221 for final report.

LL, log likelihood

| **B. Outcome: Maternal vitamin A deficiency** ^a^ | | | | | | | | | | | | | | |
| --- | --- | --- | --- | --- | --- | --- | --- | --- | --- | --- | --- | --- | --- | --- |
|  | **Model 1** | **Model 2** | **Model 3** | **Model 4** | **Model 5** | **Model 6** | **Model 7** | **Model 8** | **Model 9** | **Model 10** | **Model 11** | **Model 12** | **Model 13** | **Model 14** |
|  |  |  |  |  |  |  |  |  |  | **Optimal model** | **Lowest AIC** |  |  |  |
|  | Cattle sold | Cattle sold | Cattle sold | Cattle sold | Cattle sold | Cattle sold | Cattle sold | Cattle sold | Cattle sold | Cattle sold | x | x | x | x |
|  | Goat sold | x | x | x | x | x | x | x | x | x | x | x | x | x |
|  | Children fostered out | Children fostered out | Children fostered out | Children fostered out | Children fostered out | Children fostered out | x | x | x | x | x | x | x | x |
|  | Children living at home | Children living at home | Children living at home | Children living at home | Children living at home | Children living at home | Children living at home | Children living at home | Children living at home | Children living at home | Children living at home | Children living at home | Children living at home | Children living at home |
|  | Land size | Land size | Land size | Land size | Land size | Land size | Land size | Land size | Land size | Land size | Land size | Land size | Land size | Land size |
|  | Maternal age | Maternal age | x | x | x | x | x | x | x | x | x | x | Maternal age | x |
|  | Infant age | Infant age | Infant age | x | x | x | x | x | x | x | x | x | x | Infant age |
|  | Community | Community | Community | Community | Community | Community | Community | Community | Community | x | x | x | x | x |
|  | Inflammation | Inflammation | Inflammation | Inflammation | Inflammation | Inflammation | Inflammation | Inflammation | Inflammation | Inflammation | Inflammation | x | Inflammation | Inflammation |
|  | Parity | Parity | Parity | Parity | Parity | x | x | x | x | x | x | x | x | x |
|  | Household size | Household size | Household size | Household size | Household size | Household size | Household size | Household size | x | x | x | x | x | x |
|  | Complementary feeding | Complementary feeding | Complementary feeding | Complementary feeding | x | x | x | x | x | x | x | x | x | x |
|  | Breast feeding freq. | Breast feeding freq. | Breast feeding freq. | Breast feeding freq. | Breast feeding freq. | Breast feeding freq. | Breast feeding freq. | x | x | x | x | x | x | x |
| **AIC** | 187.0 | 181.7 | 179.7 | 178.1 | 176.3 | 174.8 | 173.9 | 173.1 | 172.4 | 170.9 | 168.9 | 170.1 | 170.8 | 170.9 |
| **∆AIC** | 18.2 | 12.8 | 10.8 | 9.2 | 7.4 | 6.0 | 5.1 | 4.2 | 3.6 | 2.1 | 0.0 | 1.3 | 1.9 | 2.0 |
| **BIC** | 251.4 | 235.9 | 230.5 | 225.5 | 220.3 | 215.5 | 211.2 | 207.0 | 202.9 | 194.7 | 182.4 | 180.3 | 187.7 | 187.8 |
| **LL** | -74.5 | -74.8 | -74.9 | -75.0 | -75.1 | -75.4 | -76.0 | -76.5 | -77.2 | -78.5 | -80.4 | -82.1 | -80.4 | -80.4 |
| **n** | 219 | 219 | 219 | 219 | 219 | 219 | 219 | 219 | 219 | 219 | 219 | 219 | 219 | 219 |

^a^ Model selection was done restricting the sample size to n 219 for fair comparisons of AIC values across nested models. The selected models were then rerun for n 220 for final report.

LL, log likelihood

| **C. Outcome: Hyperhomocysteinemia ^a^** | | | | | | | | | | | | | | | | |
| --- | --- | --- | --- | --- | --- | --- | --- | --- | --- | --- | --- | --- | --- | --- | --- | --- |
|  | **Model 1** | **Model 2** | **Model 3** | **Model 4** | **Model 5** | **Model 6** | **Model 7** | **Model 8** | **Model 9** | **Model 10** | **Model 11** | **Model 12** | **Model 13 Lowest AIC** | **Model 14** | **Model 15 Optimal model** | **Model 16 Best model ^b^** |
|  |  |  |  |  |  |  |  |  |  |  |  |  |  |  |  |  |
|  | Cattle sold | Cattle sold | Cattle sold | Cattle sold | Cattle sold | Cattle sold | Cattle sold | Cattle sold | Cattle sold | Cattle sold | x | Cattle sold | Cattle sold | Cattle sold | x | x |
|  | Goat sold | Goat sold | Goat sold | Goat sold | Goat sold | Goat sold | Goat sold | Goat sold | Goat sold | Goat sold | Goat sold | Goat sold | Goat sold | x | Goat sold | x |
|  | Children fostered out | Children fostered out | Children fostered out | Children fostered out | Children fostered out | Children fostered out | Children fostered out | Children fostered out | Children fostered out | Children fostered out | Children fostered out | Children fostered out | Children fostered out | Children fostered out | Children fostered out | Children fostered out |
|  | Children living at home | Children living at home | Children living at home | Children living at home | Children living at home | Children living at home | Children living at home | Children living at home | Children living at home | x | Children living at home | Children living at home | x | x | x | x |
|  | Land size | Land size | Land size | x | x | x | x | x | x | x | x | x | x | x | x | x |
|  | Maternal age | Maternal age | x | x | x | x | x | x | x | x | x | x | x | x | x | x |
|  | Infant age | x | x | x | x | x | x | x | x | x | x | x | x | x | x | x |
|  | Community | Community | Community | Community | Community | Community | Community | Community | Community | Community | Community | Community | Community | Community | Community | Community |
|  | Inflammation | Inflammation | Inflammation | Inflammation | Inflammation | Inflammation | x | x | x | x | x | x | x | x | x | x |
|  | Parity | Parity | Parity | Parity | Parity | Parity | Parity | Parity | x | Parity | Parity | x | x | x | x | x |
|  | Household size | Household size | Household size | Household size | x | x | x | x | x | x | x | x | x | x | x | x |
|  | Complementary feeding | Complementary feeding | Complementary feeding | Complementary feeding | Complementary feeding | x | x | x | x | x | x | x | x | x | x | x |
|  | Breast feeding freq. | Breast feeding freq. | Breast feeding freq. | Breast feeding freq. | Breast feeding freq. | Breast feeding freq. | Breast feeding freq. | x | x | x | x | x | x | x | x | x |
| **AIC** | 130.4 | 128.4 | 126.6 | 124.9 | 123.2 | 121.5 | 120.4 | 119.7 | 120.5 | 121.1 | 121.4 | 120.5 | 119.1 | 119.2 | 120.1 | 119.2 |
| **∆AIC** | 11.3 | 9.3 | 7.5 | 5.8 | 4.1 | 2.4 | 1.3 | 0.6 | 1.4 | 2 | 2.3 | 1.4 | 0 | 0.1 | 1 | 0.1 |
| **BIC** | 185.8 | 180.7 | 175.8 | 171.1 | 166.3 | 161.5 | 157.3 | 153.6 | 151.3 | 151.8 | 149.9 | 151.3 | 137.7 | 137.7 | 142.3 | 131.8 |
| **LL** | -47.2 | -47.2 | -47.3 | -47.5 | -47.6 | -47.8 | -48.2 | -48.9 | -50.3 | -50.5 | -51.7 | -50.3 | -50.6 | -53.6 | -53.1 | -55.6 |
| **n** | 160 | 160 | 160 | 160 | 160 | 160 | 160 | 160 | 160 | 160 | 175 | 160 | 160 | 160 | 175 | 175 |

^a^ Model selection was done restricting the sample size to n 175 for fair comparisons of AIC values across nested models. The selected models were then rerun for n 176 for final report.

^b^ Model 16 was selected as the best model although model 13 had the lowest AIC because model 16 had the combination of low AIC (within 2 of the lowest) and the large sample size. Fifteen observations in the ≤50% cattle sold category predicted normal homocysteine perfectly and were therefore dropped. LL, log likelihood

Table S2 Final GSEM models for maternal malnutrition and milk variables

## a) GSEM model for maternal underweight and milk energy

| Variable | Path  Coefficient | 90% CI | *P*-value |
| --- | --- | --- | --- |
| ***Response: Maternal underweight (n 221)*** |  |  |  |
| Children fostered out ^a^ | -1.05 | -1.70, -0.40 | 0.008 |
| *z* Land size ^b^ | 0.36 | 0.10, 0.61 | 0.021 |
| Cattle sold |  |  |  |
| None sold (Ref.) | - | - | - |
| No animal | -0.25 | -0.87, 0.38 | 0.514 |
| >50% sold | -1.08 | -2.01, -0.14 | 0.059 |
| ≤50% sold | -0.53 | -1.58, 0.53 | 0.412 |
| *z* Infant age ^b^ | 0.32 | 0.06, 0.57 | 0.043 |
| Inflammation | 0.67 | 0.03, 1.32 | 0.087 |
| Community |  |  |  |
| Karare (Ref.) | - | - | - |
| Kituruni | -0.67 | -1.46, 0.11 | 0.159 |
| Korr | 0.67 | -0.06, 1.41 | 0.132 |
|  |  |  |  |
| ***Response: z ln milk energy (n 202)*** |  |  |  |
| Maternal underweight | -0.12 | -0.36, 0.13 | 0.431 |
| Community |  |  |  |
| Karare (Ref.) | - | - | - |
| Kituruni | 0.13 | -0.17, 0.43 | 0.466 |
| Korr | 0.36 | 0.01, 0.70 | 0.092 |

Log likelihood -410.56, AIC 851.13, BIC 902.20

^a^ ordinal variable: 0, 1, and ≥2 biological children of participants living elsewhere

^b^ standardized variables were used

## b) GSEM models for maternal vitamin A deficiency and milk retinol

|  | Model A | | | Model B | | | Model C | | |
| --- | --- | --- | --- | --- | --- | --- | --- | --- | --- |
| Variable | Path Coefficient | 90% CI | P-Value | Path Coefficient | 90% CI | P-Value | Path Coefficient | 90% CI | P-Value |
| ***Response: Maternal vitamin A deficiency (n 220)*** |  |  |  |  |  |  |  |  |  |
| Children at home | -0.24 | -0.44, -0.04 | 0.054 | -0.26 | -0.47, -0.04 | 0.046 | -0.42 | -1.09, 0.26 | 0.308 |
| *z* Land size ^a^ | 0.34 | 0.07, 0.62 | 0.041 | 1.54 | 0.58, 2.51 | 0.009 | 0.40 | 0.12, 0.69 | 0.020 |
| Cattle sold |  |  |  |  |  |  |  |  |  |
| None sold (Ref.) | - | - | - | - | - | - | - | - | - |
| No animal | 1.13 | 0.04, 2.22 | 0.089 | 1.21 | 0.10, 2.31 | 0.074 | 1.15 | -0.93, 3.23 | 0.364 |
| >50% sold | 1.11 | -0.19, 2.41 | 0.160 | 1.11 | -0.22, 2.45 | 0.170 | 0.58 | -1.75, 2.91 | 0.683 |
| ≤50% sold | 1.22 | -0.26, 2.70 | 0.175 | 1.23 | -0.28, 2.74 | 0.180 | -3.24 | -7.62, 1.13 | 0.223 |
| Children living at home × *z* land size |  |  |  | -0.36 | -0.66, -0.06 | 0.052 |  |  |  |
| Children at home × Cattle sold |  |  |  |  |  |  |  |  |  |
| None sold (Ref.) |  |  |  |  |  |  | - | - | - |
| No animal |  |  |  |  |  |  | -0.00 | -0.74, 0.73 | 0.998 |
| >50% sold |  |  |  |  |  |  | 0.22 | -0.57, 1.02 | 0.648 |
| ≤50% sold |  |  |  |  |  |  | 1.13 | 0.13, 2.13 | 0.064 |
| Inflammation | 0.95 | 0.17, 1.72 | 0.044 | 0.97 | 0.18, 1.75 | 0.043 | 1.10 | 0.30, 1.91 | 0.024 |
|  |  |  |  |  |  |  |  |  |  |
| ***Response: z ln milk retinol (n 173)*** |  |  |  |  |  |  |  |  |  |
| Children at home | 0.10 | 0.04, 0.16 | 0.007 | 0.95 | 0.03, 0.16 | 0.012 | 0.28 | 0.13, 0.43 | 0.002 |
| *z* Land size ^a^ |  |  |  | 0.34 | 0.03, 0.65 | 0.075 |  |  |  |
| Cattle sold |  |  |  |  |  |  |  |  |  |
| None sold (Ref.) |  |  |  |  |  |  | - | - | - |
| No animal |  |  |  |  |  |  | 0.41 | -0.18, 1.01 | 0.255 |
| >50% sold |  |  |  |  |  |  | 0.45 | -0.28, 1.18 | 0.317 |
| ≤50% sold |  |  |  |  |  |  | 1.05 | -0.00, 2.10 | 0.100 |
| Children at home × *z* land size |  |  |  | -0.07 | -0.13, -0.00 | 0.094 |  |  |  |
| Children at home × Cattle sold |  |  |  |  |  |  |  |  |  |
| None sold (Ref.) |  |  |  |  |  |  | - | - | - |
| No animal |  |  |  |  |  |  | -0.21 | -0.42, -0.03 | 0.044 |
| >50% sold |  |  |  |  |  |  | -0.18 | -0.38, 0.02 | 0.139 |
| ≤50% sold |  |  |  |  |  |  | -0.32 | -0.58, -0.06 | 0.043 |
| Maternal vitamin A deficiency | -0.03 | -0.38, 0.33 | 0.90 | -0.12 | -0.48, 0.25 | 0.597 | 0.04 | -0.39, 0.47 | 0.856 |

Log Likelihood, AIC, and BIC for Model A -320.40, 662.80, and 700.13; Model B -315.97, 659.94, and 707.45; Model C -313.40, 666.81, and 734.68

^a^ standardized variables were used

## c) GSEM model for maternal hyperhomocysteinemia and milk folate receptor-α

| Variable | Path Coefficient | 90% CI | P-Value |
| --- | --- | --- | --- |
| ***Response: Maternal Hyperhomocysteinemia (n 176)*** |  |  |  |
| Children fostered out ^a^ | 0.67 | 0.04, 1.30 | 0.080 |
| Goats/sheep sold |  |  |  |
| None sold (Ref.) |  |  |  |
| No animal to sell | 1.27 | 0.02, 2.51 | 0.095 |
| >50% sold | 1.69 | 0.26, 3.13 | 0.052 |
| ≤50% sold | 0.74 | -0.75, 2.23 | 0.412 |
| Community |  |  |  |
| Karare (Ref.) | - | - | - |
| Kituruni | -1.24 | -3.02, 0.54 | 0.250 |
| Korr | 1.52 | 0.44, 2.59 | 0.021 |
|  |  |  |  |
| ***Response: z ln milk retinol (n 176)*** |  |  |  |
| Maternal hyperhomocysteinemia | 0.34 | 0.00, 0.75 | 0.099 |
| Community |  |  |  |
| Karare (Ref.) |  |  |  |
| Kituruni | -0.39 | -0.70, -0.08 | 0.037 |
| Korr | 0.41 | 0.07, 0.076 | 0.049 |

Log Likelihood, AIC, and BIC: -289.01, 602.02, 640.07

^a^ ordinal variable: 0, 1, and ≥2 biological children of participants living elsewhere

## Table S3 GSEM models for maternal nutritional and milk variables using the alternative children fostered out

## GSEM model for maternal underweight and milk energy

| Variable | Path  Coefficient | 90% CI | *P*-value |
| --- | --- | --- | --- |
| ***Response: Maternal underweight (n 221)*** |  |  |  |
| Children fostered out ^a^ | -0.71 | -1.37, -0.05 | 0.077 |
| *z* Land size ^b^ | 0.31 | 0.06, 0.56 | 0.038 |
| Cattle sold |  |  |  |
| None sold (Ref.) | - | - | - |
| No animal | -0.30 | -0.92, 0.32 | 0.431 |
| >50% sold | -1.07 | -2.00, -0.14 | 0.058 |
| ≤50% sold | -0.46 | -1.51, 0.58 | 0.466 |
| *z* Infant age ^b^ | 0.32 | 0.07, 0.57 | 0.038 |
| Inflammation | 0.63 | -0.01, 1.26 | 0.104 |
| Community |  |  |  |
| Karare (Ref.) | - | - | - |
| Kituruni | -0.66 | -1.43, 0.11 | 0.156 |
| Korr | 0.67 | -0.06, 1.40 | 0.128 |
|  |  |  |  |
| ***Response: z ln milk energy (n 202)*** |  |  |  |
| Maternal underweight | -0.12 | -0.36, 0.13 | 0.431 |
| Community |  |  |  |
| Karare (Ref.) | - | - | - |
| Kituruni | 0.13 | -0.17, 0.43 | 0.466 |
| Korr | 0.36 | 0.01, 0.70 | 0.092 |

Log Likelihood: -289.01

^a^ ordinal variable: 0, 1, and ≥2 biological children of participants living elsewhere

Female children 16 years or older and living elsewhere were recoded as married.

## GSEM model for maternal hyperhomocysteinemia and milk folate receptor-α using the alternative children fostered out

| Variable | Path Coefficient | 90% CI | P-Value |
| --- | --- | --- | --- |
| ***Response: Maternal Hyperhomocysteinemia (n 176)*** |  |  |  |
| Children fostered out ^a^ | 0.79 | 0.12, 1.45 | 0.052 |
| Goats/sheep sold |  |  |  |
| None sold (Ref.) |  |  |  |
| No animal to sell | 1.25 | 0.003, 2.50 | 0.099 |
| >50% sold | 1.68 | 0.25, 3.12 | 0.054 |
| ≤50% sold | 0.71 | -0.78, 2.20 | 0.432 |
| Community |  |  |  |
| Karare (Ref.) | - | - | - |
| Kituruni | -1.23 | -3.01, 0.55 | 0.256 |
| Korr | 1.51 | 0.43, 2.59 | 0.022 |
|  |  |  |  |
| ***Response: z ln milk folate receptor- α (n 176)*** |  |  |  |
| Maternal hyperhomocysteinemia | 0.38 | 0.00, 0.75 | 0.099 |
| Community |  |  |  |
| Karare (Ref.) |  |  |  |
| Kituruni | -0.39 | -0.70, -0.08 | 0.037 |
| Korr | 0.41 | 0.07, 0.76 | 0.049 |

Log Likelihood: -289.01

^a^ ordinal variable: 0, 1, and ≥2 biological children of participants living elsewhere.

Female children 16 years or older and living elsewhere were recoded as married.
